# THERAPIST: Towards an Autonomous Socially Interactive Robot for Motor and Neurorehabilitation Therapies for Children

**DOI:** 10.2196/rehab.3151

**Published:** 2014-10-07

**Authors:** Luis Vicente Calderita, Luis J Manso, Pablo Bustos, Cristina Suárez-Mejías, Fernando Fernández, Antonio Bandera

**Affiliations:** ^1^RoboLab, University of ExtremaduraCáceresSpain; ^2^Technological Innovation Group, Virgen del Rocío University HospitalSevilleSpain; ^3^Computer Science Department, University Carlos IIIMadridSpain; ^4^Departamento de Tecnología Electrónica, University of MálagaMálagaSpain

**Keywords:** rehabilitation, cognitive robotics, interactive games

## Abstract

**Background:**

Neurorehabilitation therapies exploiting the use-dependent plasticity of our neuromuscular system are devised to help patients who suffer from injuries or diseases of this system. These therapies take advantage of the fact that the motor activity alters the properties of our neurons and muscles, including the pattern of their connectivity, and thus their functionality. Hence, a sensor-motor treatment where patients makes certain movements will help them (re)learn how to move the affected body parts. But these traditional rehabilitation processes are usually repetitive and lengthy, reducing motivation and adherence to the treatment, and thus limiting the benefits for the patients.

**Objective:**

Our goal was to create innovative neurorehabilitation therapies based on THERAPIST, a socially assistive robot. THERAPIST is an autonomous robot that is able to find and execute plans and adapt them to new situations in real-time. The software architecture of THERAPIST monitors and determines the course of action, learns from previous experiences, and interacts with people using verbal and non-verbal channels. THERAPIST can increase the adherence of the patient to the sessions using serious games. Data are recorded and can be used to tailor patient sessions.

**Methods:**

We hypothesized that pediatric patients would engage better in a therapeutic non-physical interaction with a robot, facilitating the design of new therapies to improve patient motivation. We propose RoboCog, a novel cognitive architecture. This architecture will enhance the effectiveness and time-of-response of complex multi-degree-of-freedom robots designed to collaborate with humans, combining two core elements: a deep and hybrid representation of the current state, own, and observed; and a set of task-dependent planners, working at different levels of abstraction but connected to this central representation through a common interface. Using RoboCog, THERAPIST engages the human partner in an active interactive process. But RoboCog also endows the robot with abilities for high-level planning, monitoring, and learning. Thus, THERAPIST engages the patient through different games or activities, and adapts the session to each individual.

**Results:**

RoboCog successfully integrates a deliberative planner with a set of modules working at situational or sensorimotor levels. This architecture also allows THERAPIST to deliver responses at a human rate. The synchronization of the multiple interaction modalities results from a unique scene representation or model. THERAPIST is now a socially interactive robot that, instead of reproducing the phrases or gestures that the developers decide, maintains a dialogue and autonomously generate gestures or expressions. THERAPIST is able to play simple games with human partners, which requires humans to perform certain movements, and also to capture the human motion, for later analysis by clinic specialists.

**Conclusions:**

The initial hypothesis was validated by our experimental studies showing that interaction with the robot results in highly attentive and collaborative attitudes in pediatric patients. We also verified that RoboCog allows the robot to interact with patients at human rates. However, there remain many issues to overcome. The development of novel hands-off rehabilitation therapies will require the intersection of multiple challenging directions of research that we are currently exploring.

## Introduction

Neurorehabilitation therapy pursues the recovery of damaged neuronal areas and/or muscles from the repetitive practice of certain motor or cognitive activities. The patient’s recovery directly depends on the adherence to the neurorehabilitation therapy. Conventional methods consisting of repetitions usually make the patient feel unmotivated, and they neglect to comply with the appropriate treatments. In addition, the treatment of these motor and cognitive deficits requires intensive and extended rehabilitation sessions that demand sustained dedication and effort by professionals and incur additional costs for the institutions. In recent years, robotic science has become a useful tool to address these issues. For instance, the SCRIPT project [1], funded by the European Union 7th Frame Program, includes a work package for motivational rehabilitation. The aim is to develop novel techniques to guide and encourage patients, ensuring that the process is as intuitive and interesting as possible. As other initiatives, the SCRIPT project focuses on telerobotic procedures, with a strategy that guarantees safety and robustness in a critical scenario but bounds the robot’s autonomy. With the aim of increasing this autonomy, one of the most active research fields in this area is the design of socially assistive robots [2]. These robots can be used in non-contact, hands-off therapeutic interactions with the patient, exploiting embodiment, emotions, dialogues, personality, user models, and socially situated learning. They may provide cost-effective solutions to the need of extended and dedicated one-on-one care and also monitor progress during physical therapy and daily life, providing tireless motivation, encouragement, and guidance.

From pioneering systems such as Java Therapy [3], the application of computer-assisted technologies for rehabilitation has generated positive feedback from therapists and an increasing demand for solutions that will put the emphasis on motivation through entertainment. For instance, the ArmeoSpring Pediatric from Hocoma [4] is a robotic tool to improve therapy by facilitating intensive and functional movement exercises. This tool supports the therapy by motivating game-like tasks. However, given the inherent human tendency to engage with life-like social behavior, the use of the robot for augmenting or maintaining the patient’s motivation provides an important advantage over game-based approaches [5].

This paper describes our own experience with the design and development of THERAPIST, a robot for hands-off interaction that allows the definition of new neurorehabilitation therapies. In this kind of application, the role of physical embodiment, the capacity of responding to new events, and the ability for making use of multiple hands-off interaction strategies (eg, speech or facial expressions) are fundamental topics. In order to fulfil these requirements, we argue that the robot should be endowed with a robust and efficient cognitive architecture that guarantees autonomy and safety. To this end, we focus on describing RoboCog, a new cognitive software architecture that allows THERAPIST to perform as an innovative trainer in motor deficit therapies. THERAPIST is a socially interactive robot endowed with the necessary cognitive functionalities to allow it to operate as an autonomous, active assistant. To achieve these social behaviors that make human-robot interaction efficient and friendly, the cognitive architecture that RoboCog is based on is the internalization of the perceived information coming from multiple sources within a synchronized inner model. The higher, symbolic level of this internal representation is also available to a decision-making framework that endows the architecture with high-level planning, monitoring, learning, and re-planning abilities.

In this paper, we describe the motivation and main guidelines of RoboCog, the internal cognitive architecture for THERAPIST, experimental tests and results, and future work.

##  Methods

### From Ursus to Therapist: Toward a Socially Assistive Robot

**Figure 1:**
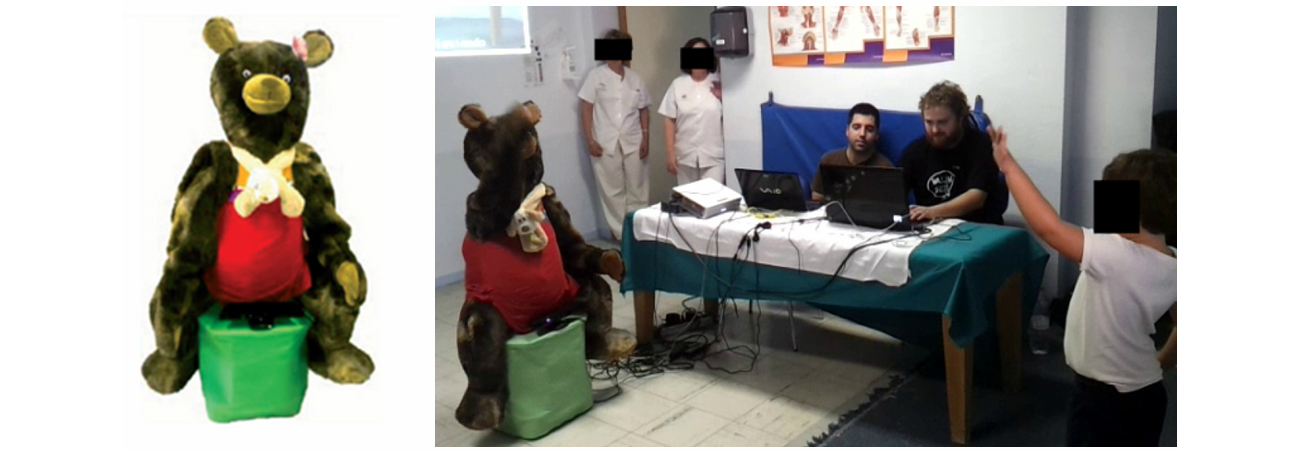
(Left) The robot URSUS and (Right) URSUS driving a rehabilitation session at the Hospital Universitario Virgen del Rocío (Seville).

In 2009, the project ACROSS [6] was launched. It was a Singular Strategic Scientific-Technological project funded by the Spanish government under the Plan Avanza initiative whose main aim was to incorporate social robots in actual human-robot interaction scenarios. In this project, research groups from the University of Extremadura (RoboLab, UEx) and of the Hospital Universitario Virgen del Rocío (HUVR) worked together on the development of a robot that helps patients and therapists in the execution of repetitive rehabilitation exercises. The robot was named URSUS (see Figure 1). It is a semi-autonomous robot, equipped with a Color (Red-Green-Blue) and Depth Camera (RGBD) sensor that monitors the patient’s movements. Although URSUS has proven that the predisposition of the patient to their neurorehabilitation treatments can be improved [7], its internal control architecture needs to be redefined at all abstraction levels. Thus, on one hand, it encodes each therapeutic session as a fixed hierarchical state machine. As this machine cannot cope with the new situations that continuously appear during sessions, the robot must work under human supervision. Furthermore, an easy way to learn from these situations does not exist. On the other hand, due to the inherent tendency of humans to personify animated objects [8], URSUS does not have the skills to always meet the expectations of patients and caregivers, which generates some degree of frustration. Finally, we needed to take into account several considerations about the physical appearance of URSUS, since recent studies have already demonstrated that the size of the robots has a considerable impact on the perception of their role and interaction skills [8].

Although physical issues will also be reconsidered in the design of THERAPIST, we are currently working on the development and validation of the software architecture. The RoboCog architecture is based on cognitive principles. Briefly, cognition is the ability that allows us to internally deal with the information about ourselves and the external world. Clearly, this ability is subject to the existence of an internal active representation handling all this information. The existence of a deep, hybrid inner representation of the outer world is the key concept of RoboCog. When making decisions that directly involve human users, the traditional three-tier planning and plan execution scheme [9], which separates symbolic high-level planning from geometric plan execution, is not the best strategy [10]. The generation of symbolic plans can be relatively slow, thus the approach has to rely on an (almost) static world. Such an assumption is not only unrealistic but also produces behavior that does not react to changes, which feels unnatural to humans. Contrary to these approaches, the world model used in RoboCog provides a way to synchronize the behavior of software components working at different abstraction layers.

As shown in the next section, the architecture provides a common interface to provide access to the inner model to all modules in the robot’s software. This assures the synchronization of the internalization of the outer world and a close relationship between perception and action. Furthermore, it allows multiple high- and mid-level modules to run in parallel, so the robot can provide fast responses to new situations. As in other behavior-based control architectures, low- and mid-level components are always active [11]. High-level components are also active continuously, allowing the correct planning and monitoring of the course of action. This plan is constantly updated using the information provided by the rest of the modules of the system. Experimental results show that THERAPIST is able to establish a natural interaction with pediatric patients. Endowed with RoboCog, THERAPIST is currently an effective socially assistive robot [8].

### RoboCog, the Software Architecture of THERAPIST

**Figure 2:**
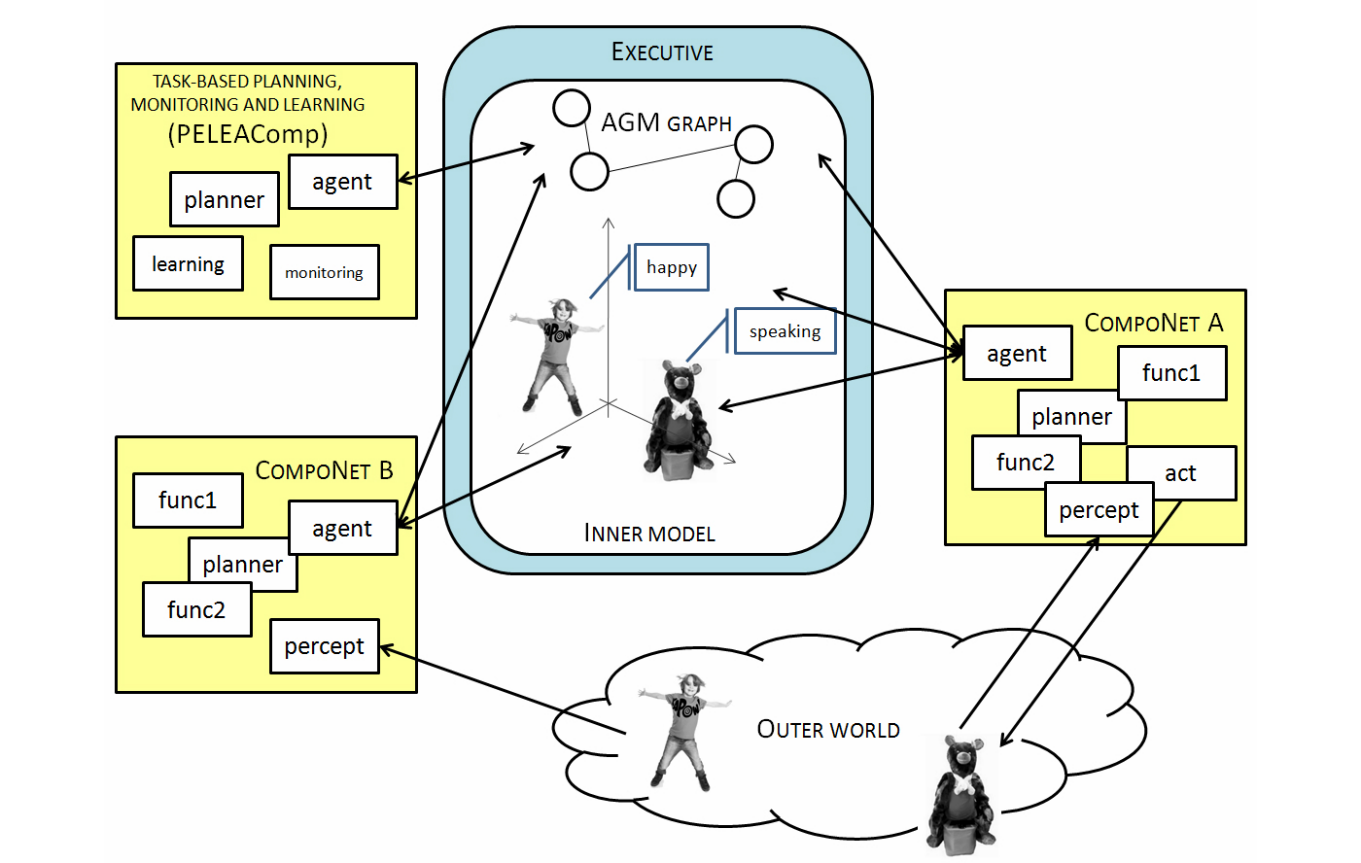
An overview of the RoboCog.

Figure 2 shows a schematic overview of the RoboCog cognitive architecture. The internal representation (Inner Model) is hierarchically organized, providing different interfaces at levels of abstraction that range from the fine-grained aspects of motor control to the symbolic ones needed by the rational control. The existence of this deep, hybrid representation for action, perception, and emulation is the main novelty of the architecture. The concept of deep representation is clearly described by Beetz et al [12]: “representations that combine various levels of abstraction, ranging, for example, from the continuous limb motions required to perform an activity to atomic high-level actions, subactivities, and activities”. But the concept of deep representations, as described by Beetz et al, implies a unified, hierarchical representation of the robot. In our framework, this definition should be extended to consider representation and inference mechanisms for models including the person’s body, actions, abilities, and intentions. At a high level of abstraction, our representation manages information related to the person’s activity and degree of interest but also with the robot’s activity. The inclusion of a detailed physical layer on the representation will allow the robot to solve naive physical problems that are not well suited to classical logic, using temporal projection [13].

The RoboCog architecture has three main elements (Figure 2). The internal representation of the environment (Inner Model) and the Executive represent the core of the architecture. This core interacts with the planning monitoring and learning module, and with a series of networks of task-oriented software components in charge of active and perceptual tasks. These task-oriented networks, called compoNets, are connected to the different levels of abstraction of the Inner Model through special components called agents. In our architecture, the Inner Model is a graph where information is stored at different levels of abstraction. At the higher level of abstraction, this graph contains a set of nodes and arcs attributed with symbolic information. These items constitute an AGM (Active Grammar-based Modeling) graph [14], which stores the information required for deliberative planners. Linked to these symbolic nodes, the Inner Model stores the sensorimotor information on a kinematic tree. This lower level of abstraction includes the world and the items on it (robot, objects, and people). Between the symbolic and sensorimotor levels of abstraction, we can also define a situational level by annotating the nodes of the kinematic tree with additional attributes (eg, detailing the patient speaking). There are significant differences in the way that the three structures encoded within the Inner Model process the stored information. Thus, the AGM graph changes according to a grammar, so that updates can be validated. On the contrary, the situational attributes on the kinematic tree can be updated by the agents in a faster way. The Executive module manages the Inner Model: it publishes the graph’s updates to the compoNets and receives the modification proposals provided by the compoNets as they perform actions or detect changes in the environment. Furthermore, the Executive filters the information related to the AGM graph to maintain a world model representation coherent with the domain theory. So far, there is no analogous mechanism to control the updates of the kinematic tree at situational or sensorimotor levers, although some kind of filtering is locally done by the compoNets.

The Inner Model is available to a hierarchy of planners, which are included within the compoNets (Figure 2) and are in charge of achieving specific goals. According to their nature, these goals are also defined at different abstraction levels. When a mission is assigned to the robot, the Executive module is the one in charge of achieving it. This mission (overarching goal) is stated as a pattern to be found in the AGM graph. The steps needed to transform the current AGM graph so that it exactly matches the searched pattern constitute a plan. Plans are provided to the Executive by the “Task-based Planning and Monitoring” compoNet (PELEAComp) as a sequence of tasks. Given a task, the Executive is also responsible for activating the compoNets that must solve it. Within the compoNets, the agents transform the tasks into situated goals, that is, the behaviors that the compoNet must launch to solve the task (eg, maintain the interest of the person through dialogue, monitor the correct execution of a gesture, recognize facial emotions). Within each compoNet, the situated goal might require a specific planner (eg, a conversational algorithm or a path-planner) that decomposes this goal into low-level actions (sensorimotor level). The correct achievement of these low-level actions (eg, to say a phrase, to capture the movements of the arms of the patient, to capture the face, and to synthesize it using a set of features) is the responsibility of the compoNets. The aim is that, at the sensorimotor level, the compoNets manage actions that do not need interaction among several compoNets. For instance, once the sentence to be said is chosen at the situational stage (Conversational compoNet), it is sent to the Text-To-Speech (TTS) component. These low-level actions can be stopped by other components within the network when the situational goal(s) changes without focusing on the responses emanating from other compoNets. This situation differs from the one at situational or deliberative stages; for example, to speak, the robot should be looking at the child and should also be in a standing state.

Next, we provide further details about how the triadic Inner Model—Executive—PELEAComp works. We describe how the planners and the machine learning capabilities are integrated into the high-level planning, what the units of the plans are, how the Executive uses the grammar rules, and how the inner model represents models of the world that can be used to simulate and predict. The compoNets that have been integrated in RoboCog will be detailed in the Results Section, since they are specific to the proposed evaluation scenario.

###  Inner Model for Prediction, Evaluation, and Selection

In order to engage patients in social interactions, THERAPIST should be able to provide responses at human interaction rates and exhibit a pro-active behavior [15]. This pro-active behavior implies that the internal architecture of THERAPIST should be not only able to perceive and act but also to reason about the consequences of actions and how they might affect the robot’s mission. As mentioned, cognition is the ability that allows us to internalize the information about the world, and it is then subject to the existence of an internal representation of this information. Although internal models and representations of the state of the external world were traditionally rejected by the reactive paradigms [16], subsequent works suggested that cognitive architectures cannot work on a passive, bottom-up fashion, simply waiting to be activated by external stimuli [17]. Instead, these architectures must use memory to continuously interpret sensory information and predict the immediate future. For instance, these predictions about the world can be used to actively drive the resources to relevant data in top-down modes of behavior, allowing an efficient and accurate interpretation of the environment [18,19]. Without this ability, the responses of the robot in this scenario would not be generated at the needed pace, disappointing both patients and caregivers [8]. As Figure 2 shows, in our proposal, all planners can access the Inner Model. In the restricted scenario where our therapies are conducted, this model always includes the robot, a patient model, and a room. Thus, we currently have a working example of the situation described by Holland [19]: “at the heart of the mechanism is not just the body in the environment, it is a model of the body in a model of the environment”. This model is built through interaction with the outer world, and it can be used as a virtual reality scenario by all compoNets.

As mentioned, the Inner Model is organized in a hierarchical way. The symbolic level encodes the world using an AGM graph model, whose evolution is validated using a grammar-based formal method [14]. Using this representation and the high-level decision-making compoNet (PELEAComp), RoboCog provides an inherent trade-off between preconceived plans and reactive behavior. PELEAComp endows the architecture with a planning system that is also continuously learning and stores the best plans to reach a goal [20]. This learning of plans is targeted towards building a plan library, similar to early artificial intelligence planning systems [21]. To complement this ability to use previous plans, PELEAComp also offers the possibility of transforming existing plans [22]. On the other hand, the geometric level encodes the world as a kinematic tree where each relevant item (the robot, people, and the objects in the known environment) is a parent node. The whole representation can be animated by the compoNets as a virtual environment. In addition to plan transformation at a higher level, multimodal interaction between humans and robots often needs quick, last-minute adaptations due to unpredictable environment changes or human behavior. During interaction with humans, such adaptations are necessary when the conversation is interrupted or unrequired inputs are provided in the form of back-channeling agreement or disagreement, or other listener responses are shown [23]. Other aspects of bodily behavior that are difficult to plan ahead for are, for example, behavior matching and synchrony [24]. In such situations, transformational techniques based on constraint solvers where plans can be modified “on the fly” should be used [25]. The RoboCog architecture addresses this continuous interaction behavior at a situational level, that is, using attributes annotated on the nodes of the kinematic tree. These attributes are available to all compoNets and can be quickly updated if needed. As our experiments show, the situational level is able to provide the information needed to maintain a synchronized and fluent interaction with the human counterpart.

### The Executive Module and the Active Grammar-Based Modeling Graph

The Executive bridges the gap between the symbolic level (ie, the AGM graph and PELEAComp) and the compoNets. It has two main goals. On one hand, it is responsible for publishing the task to reach the compoNets. It also subscribes to the outcomes from the agents. Thus, when a compoNet perceives/provokes a change in the model (eg, when a person is detected or lost), it publishes to the Executive a tentative, new model. Using the domain knowledge, the second goal of the Executive is to check if this new model is valid. This validation occurs only when this model implies a change in the AGM graph. If the validation is positive, the whole Inner Model is changed according to the new proposal.

**Figure 3:**
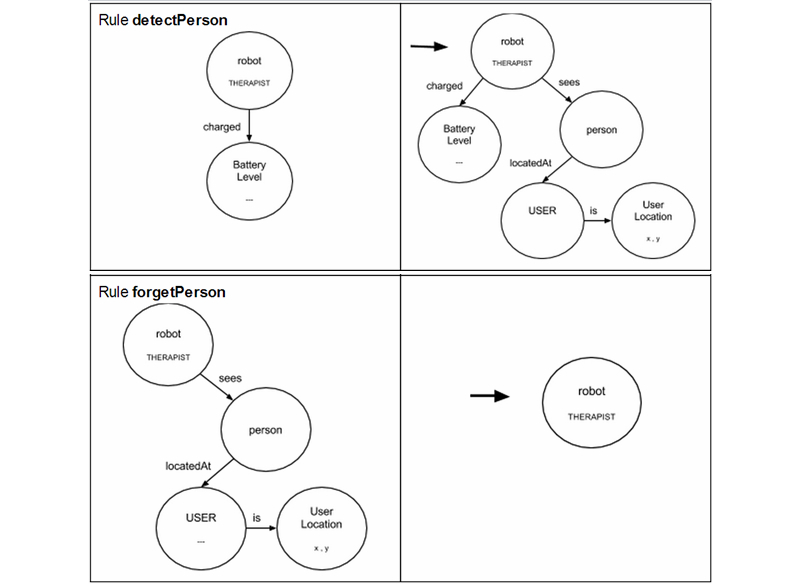
Examples of graph transformation rules used to make the robot detect or lose a person.

Both goals are related to the ability of the Executive to manage the AGM graph. Briefly, this graph allows description of the domain of the problem as a set of graph rewriting rules, equivalent to Planning Domain Definition Language (PDDL) actions (with the exception that these rules can create and delete symbols). In fact, to enable communication with PELEAComp, the rules, the current AGM model, and the robot’s goal are transparently translated to PDDL before providing them to the high-level planning system. Each rule is composed of two patterns (see Figure 3; one on the left [LHS] and one on the right [RHS]) and states that the symbolic level of the Inner Model of the robot can be modified by substituting the LHS pattern with the RHS one. Figure 3 provides two examples showing the robot detect or lose a person.

The graph grammar provides a valid tool to avoid situations that should not occur in this scenario, maintaining the consistency of the Inner Model [26]. On the contrary, it forces us to define in advance the whole set of grammatical rules that encode all the valid changes that the model can suffer. It should be noted, however, that these rules encoded on the compoNets do not represent anything other than the repertoire of capabilities provided by these same compoNets.

When the Executive receives a new task from PELEAComp, it translates this task to the compoNets. It is always publishing the current state of the AGM graph and also waiting for the responses of these compoNets, which will come in the form of tentative updates of the model. As mentioned, besides managing the Task execution, the Executive also verifies that the changes proposed by the compoNets to the AGM graph are valid by posing the verification process as a planning problem, where the initial world is the current world and the goal is the new model proposed. Modifications are considered valid if and only if the planner can find a plan to get from the former to the later world model. This verification is performed as a planning process different from the main planning process conducted by PELEAComp. The objective of PELEAComp is to provide a plan to reach the specified robot’s goals (eg, moving from location A to location B avoiding obstacles), while the verification planning process is used as a form of model checking [27].

### High-Level Planning and Monitoring compoNet: PELEAComp

#### Overview

**Figure 4:**
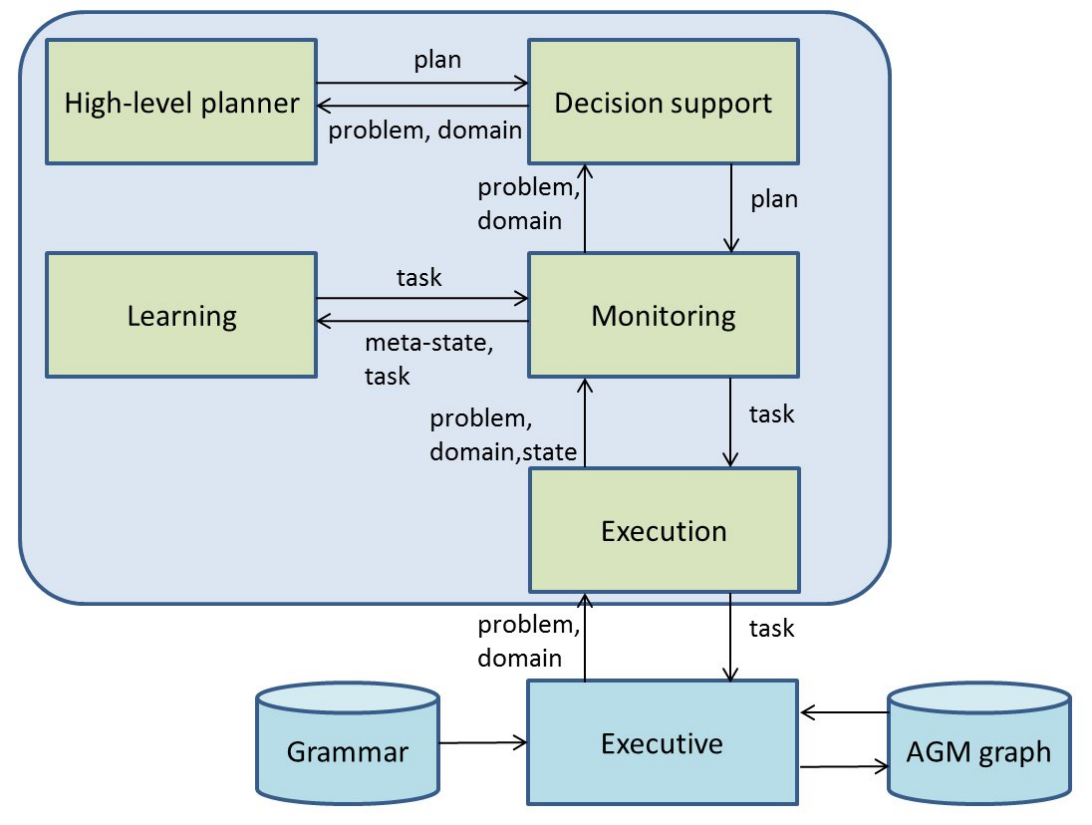
Overview of PELEAComp and its connection with the Executive module.

The high-level planning, monitoring, and learning abilities are the responsibility of the PELEAComp compoNet, an instantiation of the original PELEA framework [28]. Briefly, PELEAComp integrates planning, execution, monitoring, re-planning, and learning. Figure 4 provides an overview of PELEAComp. It is composed of four main submodules and the high-level planner.

The information shared by the submodules includes the state, an abstracted high-level, PDDL-based representation of the AGM graph; tuples (meta-state, task), learning examples to be used by the learning component to acquire knowledge for future planning episodes; the domain, a definition of the model for high-level planning; the problem, composed by the initial state and a goal to achieve; the plan, a set of ordered tasks resulting from the high-level planning process; and the task, a single unit of the plan.

Next we briefly describe each module of the architecture.

#### Monitoring Module

The Monitoring module is the main module of PELEAComp. It is responsible for checking (monitoring) the plan progress during its execution and interacting with the Execution module by receiving a problem, a domain, and a state, and returning the next high-level task to be executed. To do this, it uses the Decision Support module for a high-level plan. Additionally, it is also in charge of receiving the current world information from the Execution module. When the Monitoring module receives the new world state, it will check the expected state against this perceived world state. If some of the monitored information does not fall within the expected range, the Monitoring will have to start another planning episode to compose a new plan according to new state perceived.

#### Execution Module

The Execution module is in charge of the interaction between PELEAComp and the environment. The environment can be a software simulator (eg, MDPSim [29], widely used in the Planning community), a hardware device (robot), a software application, or a user. In this work, the Execution module handles all the communication with the Executive by an Interface Description Specific Language interface [30]. Particularly, it is responsible for initiating the work of PELEAComp by receiving a particular domain and problem to be solved and sending the high-level actions to the Executive.

#### Decision Support Module

This module is in charge of receiving the domain and problem from the Monitoring module and returning a plan by the invocation of a high-level planner. Additionally, when the Monitoring indicates a discrepancy between the observed state and the expected planning state, the Decision Support will also invoke the high-level planner to update the plan to the new state. This module can be configured to use two different planners. The first one is used for planning when there does not exist a previously generated plan. The second one is used for re-planning. In this work, Fast-Downward [31] has been selected as planner and re-planner.

#### Learning Module

This module infers knowledge from the experience gathered by the high-level planner during the plan execution. The knowledge can be used either to update the domain planning model, to improve the planning process (for instance, learning heuristics), or to reduce the task response time by the learning of a plan-based policy [20].

As a whole, the architecture works as an event-driven loop in which (1) the Executive asks PELEAComp for the optimal Task (given the current world model and goal) and activates the domain-dependent compoNets according to the Task; (2) at some point, a domain-dependent compoNet executes an action or detects an unexpected event, and notifies the Executive by proposing a change in the world model (including information about the event); and (3) the Executive verifies the change and, if valid, broadcasts the new inner representation to the rest of compoNets, re-starting the loop.

The Executive module is then responsible for receiving these tasks, such as “detect Person” or “approach Person”, and for publishing them to the compoNets. These compoNets will translate the task to situational goals, for example, the task “approach Person” tracks the patient position (Person compoNet) and approaches at interaction distance of the patient (Navigation compoNet). According to the situational goal to reach, compoNets work in several ways. They can work in a “reactive” way, by subscribing to the outcomes of certain sensors or to the state representation at the Inner Model. They can provide geometric data to update the Inner Model (eg, the Person compoNet, when it solves situational goals such as Human Motion Capturing or Face Tracking) or generate symbolic data such as “the patient is close to THERAPIST” or “the patient is getting up out of the chair”. On the other hand, these same compoNets can work in an active way, including an internal state machine or a specific planner (eg, a conversational module).

Whenever it is required by the Executive Module, PELEAComp provides the next task to execute taking into account the initial plan generated and the current state. If the current state is an expected one, PELEAComp returns the following task according to the initial plan. If the current state is unexpected (eg, because battery level has decreased quickly or the patient has disappeared suddenly), a new plan is generated (from scratch or revisiting the old one), so a new task is provided taking into account the new conditions [32].

### Emulation Abilities Within RoboCog

**Figure 5:**
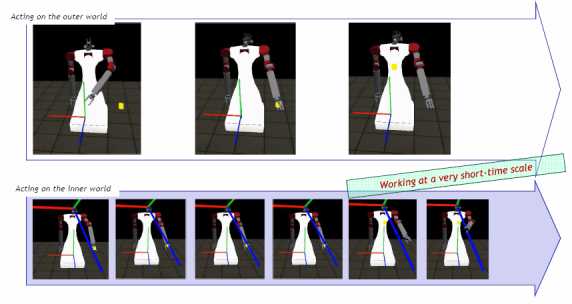
Schematic representation of the simultaneous execution of a tracking action on the outer world (top row) and within an Emulator component (bottom row). The x-axis shows the temporal axis. (The aim is that the robot touches the yellow box, and this is achieved by generating the correct motor commands for the arm).

In order to evaluate what is the best decision to reach a situational goal, a compoNet can internally emulate its action effects. For this end, the compoNet will use an emulator. The Emulator uses a copy of the model provided by the Executive to generate a simulated perception of the outcome of an action. Figure 5 shows a simple example performed inside the compoNet in charge of moving the arms of the robot. The objective is that the robot touches a yellow box. The arm is moved using a forward model instead of an inverse one, and the simulation performed at the Emulator runs faster, providing the sequence of arm motions in advance. Alternatively, Emulators can provide intermediate perceptions that allow supervision of the correct execution of a situational goal (evaluating that the sequences of changes of the model are the same for the real and the simulated execution of the action). Detected differences launch warning commands (encoded in a tentative model of the world) to the Executive and provoke a change on the course of action, that is, a re-planning step from the PELEAComp compoNet.

CompoNets can use the model to perform short-scale predictions and evaluate the consequences of actions at the situational or sensorimotor level. These processes become harder to execute as the time horizon is more distant in the future. We are currently working on translating the problem to the pure declarative domain of the AGM graph, where a symbolic automated planner can reason about the truth conditions of predicates, in a timeless, crisp representation. These symbolic systems are especially well suited for natural language understanding and interaction.

##  Results

### Implementation Details

To satisfy our requirements, the robotic platform must include many basic and advanced hardware and software components. Hence, we propose here the development of an open experimental platform for robotics software development, which has been pointed out as a necessary system from different studies [33]. This open platform will define two main elements. First, a reference low-cost mobile manipulator endowed with two robotic arms and an expressive head. As mentioned, this design improves certain features of URSUS, the robotic platform used in the clinical experiments at the Hospital Universitario Virgen del Rocío (Seville). Second, all the software modules are built over RoboComp [34], an advanced robotic framework focused on the agile design of robust components. This software platform will follow the guidelines provided by the model-driven architecture [35], allowing the platform to assist robotics engineers in the visual development, verification of real-time and QoS properties, code generation, and deployment of the required software components [36].

**Figure 6:**
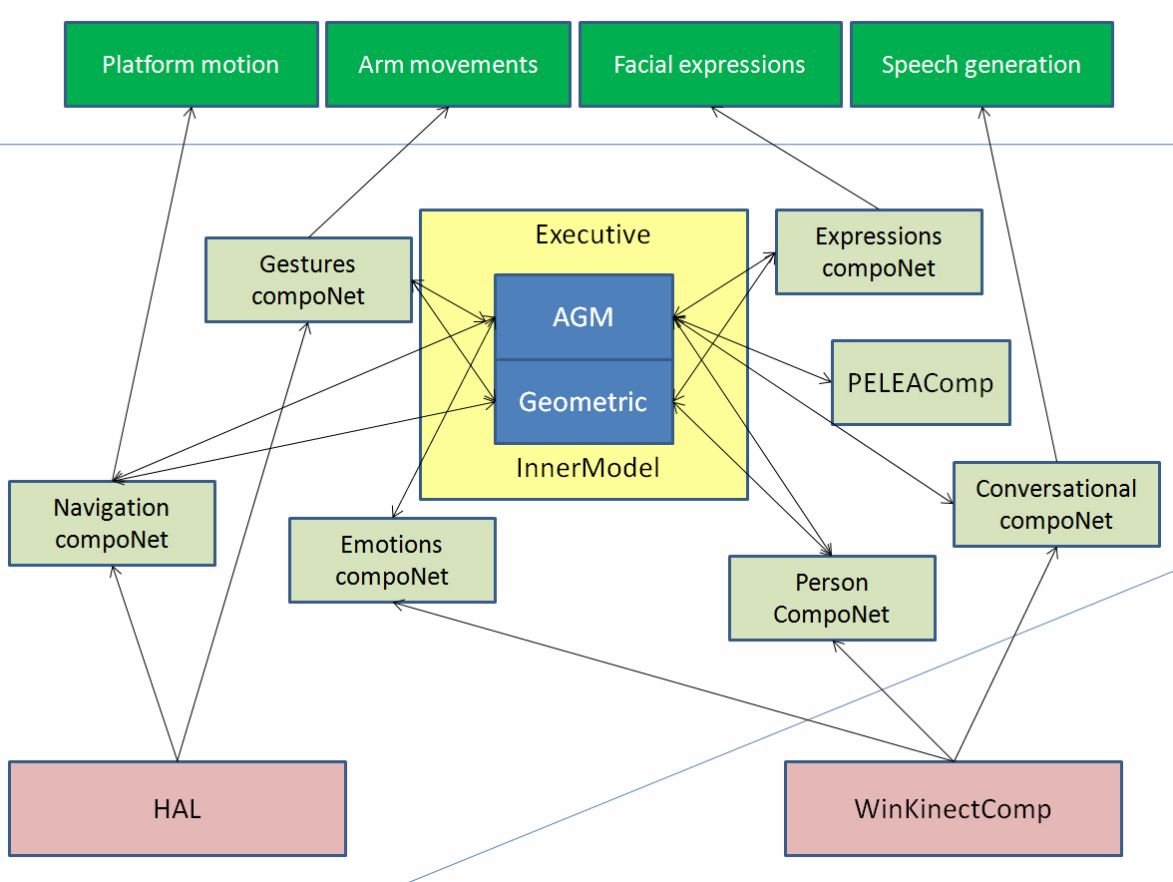
Example of the RoboCog architecture for THERAPIST.

Built over the RoboComp framework, the RoboCog architecture proposes a strategy of design and deployment of components. Figure 6 shows a block diagram of the architecture for THERAPIST. Compared to Figure 2, this new figure provides a more detailed description of modules and interfaces. The Executive and Inner Model modules will be the core of RoboCog. Together with this central representation, there are several compoNets, which interact at deliberative (AGM graph) or situational/geometric levels of abstraction. The base level of the representation is updated from the software components at the Hardware Abstraction Layer (HAL). The whole architecture is distributed on two personal computers. One of them runs on Windows operating system and is in charge of managing the data capture from a Kinect sensor (video and audio sources of information). The other one runs on Linux and manages the rest of the architecture, including software components of the HAL.

Next, we briefly describe the aim and main features of the set of modules in Figure 6. The Inner Model provides a dynamic deep representation of the robot, the patient, and the environment. As mentioned, the Executive and agents are in charge of updating the representation.

The Executive receives the Plan units (the tasks) from the PELEAComp and publishes them to the compoNets. It is also in charge of serving as the interface of the Inner Model. Thus, it publishes the state of the model to the rest of modules and receives, from these, tentative proposals of changes/updates to this model. Tentative changes to the AGM graph will be validated before being stored at the Inner Model. Transitions of the graph are stated as a set of rules. To avoid the overload of the Executive, the grammar only implements general rules, related to the managing of high-level concepts (eg, the initial detection of the patient or the detection that the patient is not showing any interest in the proposed game). Situational or sensorimotor goals are managed by the compoNets. The WinKinectComp is a specialized component whose aim is to wrap all the functionality provided by the Kinect sensor from Microsoft. The computational load of the process requires that this component runs in a second computer. It publishes to the rest of the architecture the information related to the face and body of the people in front of the robot. Furthermore, it implements the automatic speech recognition (ASR) functionality, required by the Conversational compoNet.

Then, this component implements a set of interfaces that are essential for the Person, Emotions, and Conversational compoNets: (1) MSKBodyEvent, which provides the list of bodies (with or without skeleton) in the scene and provides the list of joints of the closest skeleton (person), (2) MSKFaceEvent, which generates the position and a list of features for the faces detected by the Kinect and also provides the three-dimensional Candide grid of the closest face, and (3) MSKASR, which provides the result of the speech transcription to the Conversational component.

The communication of the WinKinectComp with the rest of RoboComp components is carried out using Ice [37]. Ice provides a native client-server communication system and a publish-subscribe event distribution service named IceStorm that decouples the connection among components. In this case, the WinKinectComp plays the publisher role while the compoNets subscribe to their publications.

The Person compoNet is responsible for the visual detection and tracking of the patient. There are two sources of information: face and body. From the face, the compoNet is able to detect where the patient is looking. From the body, the compoNet is able to refine the human motion data provided by a Kinect sensor. It should be noted that the facial emotion recognition is managed by the Emotions compoNet. Human motion capture is centered only on gestures performed by the upper limbs.

The Person compoNet is composed of two components: (1) PersonPerceptor, which subscribes to two interfaces provided by the WinKinectComp: MSKBodyEvent and MSKFaceEvent. The WinKinectComp works like a virtual sensor providing position and features of human bodies and faces. The PersonPerceptor takes all these data to generate a “person array”; and (2) PersonComp, which merges the agent-related functionalities (is in charge of proposing changes/updates to the Inner Model) with the responsibility of solving the situated goals related to this compoNet. Among other goals, the Person compoNet detects the correct pose of the patient. This detection is interesting for non-verbal interaction, but a crucial functionality for monitoring the rehabilitation exercises. The PersonComp addresses this issue using a model-based pose generator to complement the human tracking functionality provided by the WinKinectComp (that uses the Kinect SDK from Microsoft). The proposed system enforces kinematics constraints, eliminates odd poses, and filters sensor noise, while learning the real dimensions of the performer’s body. The system has been extensively tested [38].

The Conversation compoNet endows the robot with the ability to communicate with the patient through dialogue. The compoNet is connected to the WinKinectComp and the Speech Generation. The Conversational compoNet processes the information provided by the WinKinectComp, generating sentences by means of speech generation. All these processes are driven by situational goals.

**Figure 7:**
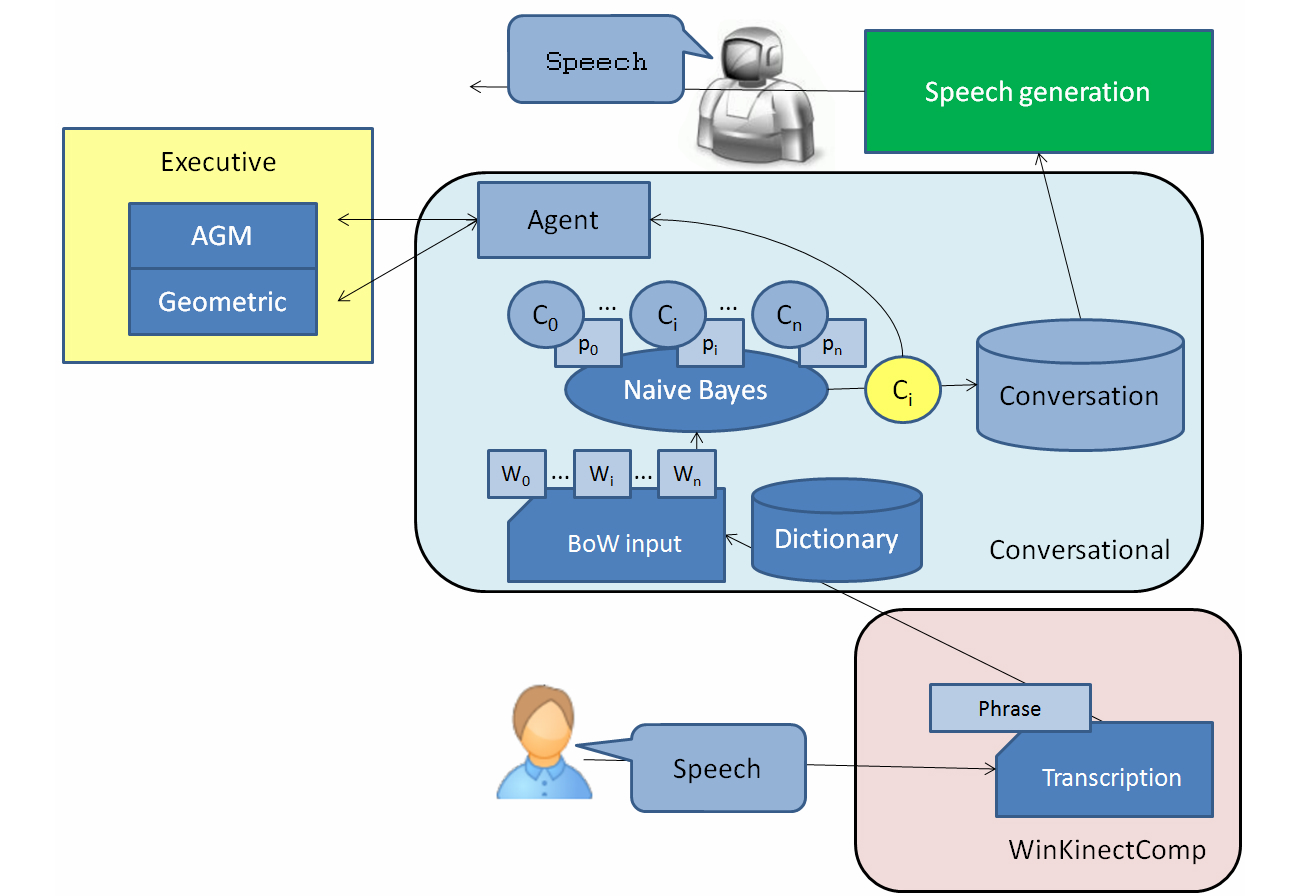
Speech recognition and generation procedure.

The speech recognition process is composed of two separate steps: transcription generation and comprehension (see Figure 7). The first step processes the audio source and obtains the most reliable text transcription. This process is completely carried out by the WinKinectComp using the Microsoft Kinect Speech SDK. Transcription generation is performed by using two internal key elements: the acoustic and the language models. The acoustic model represents the probability of obtaining an input utterance *x* given a sequence of words *w* (transcription). It is directly provided by the Microsoft Speech SDK. The language model scores the transcription *w* using the joint probability of the sequence of words. The probability for each word *w*_i_depends on the list of previous words *w*_i-1_, ... *w*_i-n_. The language model is generated by following the *n*-gram model [39], where *n* defines the number of words considered in the joint probability. The current proposal uses a 3-grams model generated from the COLA corpus [40]. Such a corpus was chosen because it includes informal ways of speaking. The 3-grams language model was then compiled using the Microsoft Speech SDK tools, obtaining a Kinect compatible grammar. The WinKinectComp uses this grammar to generate new transcriptions for each received audio input that can be modeled with the grammar. That allows environment noise to be discarded. The comprehension step is fully performed by the Conversational compoNet. It uses as input the transcription provided by the WinKinectComp and assigns it a semantic label. This semantic information is then integrated in the system, at the situational level (if the information is interesting for the HRI process) or at the deliberative level (if the information implies a change of the AGM graph).

The classification is performed using a Bag of Words (BoW) procedure [41] in conjunction with a Bayesian classifier. The dictionary for the BoW procedure is obtained with a variable selection process that removes useless words (as articles or connectors). Training and validation classifier sequences are generated using 750 sample phrases performed by more than 25 people. From these initial phrases, a grammar model is built for each one of the classes. This model includes random variations. Finally, 1800 different phrases are generated for training and 600 additional ones for validation.

When a user generates new speech, the WinKinectComp first obtains the most reliable transcription and sends it to the Conversational. If the conversation is not active (it depends on the current scenario), such transcription is discarded. Otherwise, the transcription is transformed using the BoW representation. If the obtained set of words does not include a minimum number of key words (included in the dictionary), the input phrase is directly labeled as nonsense. If not, the input set of words is processed using the Naive Bayes classifier and the set of output probabilities is studied. The input phrase is only classified as *C*_i_when *P*(*C*_i_|*w*) clearly outperforms the rest of probabilities *P*(*C*_j_|*w*)_i!=j_.

As a result of the classification, two different scenarios are identified: user question and user decision. In both situations, the compoNet requires the speech generation module to answer with an appropriate phrase. However, user decisions involve changes in the internal cognitive representation (eg, the user will be labeled as interested or not interested in playing a new game). Therefore, the Conversational can propose changes to the AGM graph through the Agent-Executive channel.

**Figure 8:**
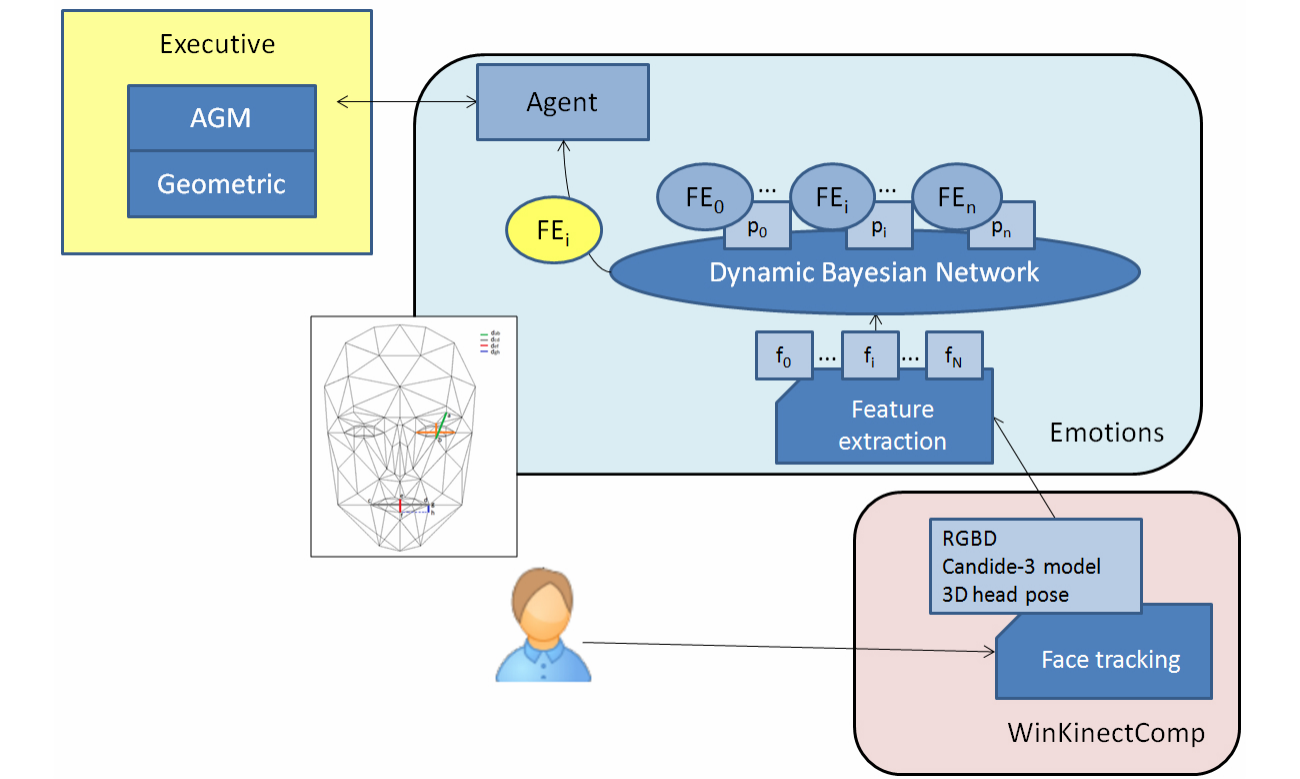
The Emotions compoNet.

The Emotions compoNet employs a Bayesian classifier to determine the emotional state of the patient based on the model of Action Units (AUs) [42]. As Figure 8 shows, the compoNet uses the grid of key points (Candide-3 model) provided by the Kinect sensor from Microsoft. From this model, the Emotions compoNet obtains the features needed to determine the facial emotion of the patient. Basically, these features are set as Euclidean distances between key points on the grid (eg, the distance between the upper contour of the eyebrows and the bottom edge of the eyes or the one between the upper contour and bottom edge of the lips). The evolution of these distances on short video sequences is directly mapped with AUs.

The dynamic Bayesian network implemented in this system uses antagonistic properties in some AUs to increase the performance and to reduce the number of variables to be considered in the network. The proposed system uses only 11 AUs, to reduce and optimize the information processing. These 11 AUs are grouped according to antagonistic and exclusive properties into only seven variables. According to Ekman’s work [42], five possible emotional states are estimated by the algorithm (ie, happy, sad, angry, fearful, and neutral). See [43] for further details about this compoNet.

The Navigation compoNet is in charge of the robot motion, including the navigation functionalities. Like the predecessor Ursus, the idea is not that THERAPIST moves from one room to another one (Figure 1 shows how Ursus is sitting on a cushion). However, to allow THERAPIST to move inside the room, the navigation compoNet uses a reactive algorithm (the R-ORM), that allows the robot to approach the patient avoiding possible obstacles.

The R-ORM algorithm is an evolution of the Obstacle Restriction Method (ORM), proposed by Chamorro and Vázquez-Martín [44]. This algorithm divides up the problem of reaching a certain objective, avoiding collisions, into a set of subproblems. Each of these subproblems consists of reaching a certain subobjective. The perceived obstacle distribution around the robot defines the positions where these subobjectives are placed. The method generates speed and turning commands that allow the robot to reach the final objective by navigating through a sequence of subobjectives.

The vanilla representation of the R-ORM has several issues when applied to real robots employed in long-term experiments and dynamic environments. The following modifications have been incorporated to the R-ORM algorithm to solve the following issues. Once R-ORM sets a subobjective, it has to reach it before computing a new one. In real experiments, this decision causes inefficient situations where a temporal occlusion of the final objective forces the selection of distant subobjectives that attract the robot even when the objective is visible again. This issue has been solved by making the robot immediately forget the current subobjective and target the final objective when it is perceived as reachable.

In order to reduce nodding produced by fast changes in the current subobjective, an inertia factor has been included. Once a new subobjective is selected, it is kept as the current goal for a certain time, which depends on the inertia value. Although R-ORM is quite robust in general, it can lead the robot to a halt position, in which it is not able to either move towards the subobjective or set a new one. A patience parameter has also been included in the code to deal with these situations. If the robot stands in the same position for a time longer than this patience value (and it has reached no objective yet), it spins for a certain time until a new subobjective is set. In the field of affective human-robot interaction (HRI), the development of robotic heads capable of generating facial expressions has come to improve the empathy and the interaction with the user.

**Figure 9:**
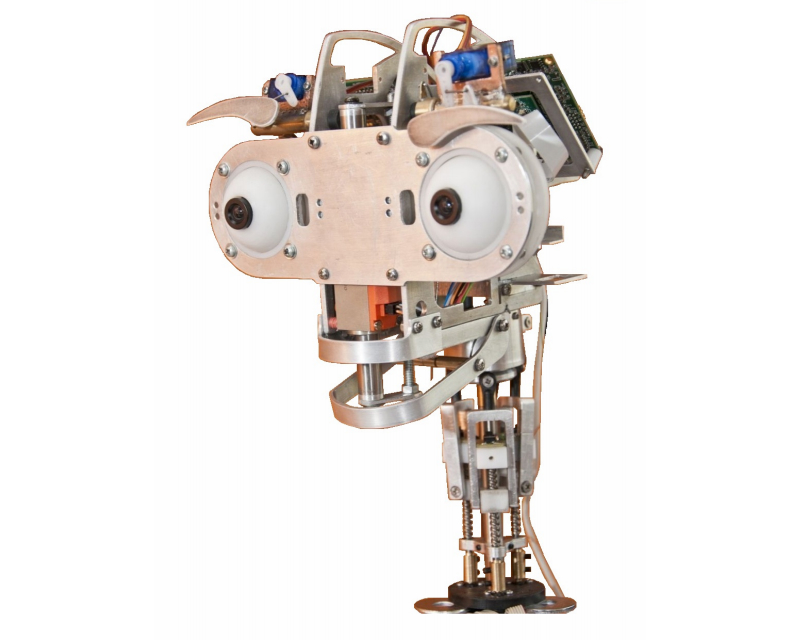
The Muecas robotic head.

In our framework, the generation of facial expressions is the responsibility of the Expressions compoNet. We can currently equip the robot with two options for its face: the robotic head Muecas [43] and a simple tablet. Muecas (Figure 9) has 12 degrees of freedom that are distributed as follows: neck (four), mouth (one), eyes (three), and eyebrow (four). The design requirements of Muecas aim to combine these mobile elements with sensors to establish an affective HRI through a human-like appearance and the use of facial expressions. The advantages of the use of a real robotic face instead of a two-dimensional representation on a planar monitor were evaluated within our group [45]. However, we do not discard its final use because of the decreasing computational complexity and, mainly, on price. Both options are able to generate and imitate emotions [43,45].

The Expressions compoNet is also in charge of synchronizing the speech with lip motion (lip-syncing). To do that, we use an algorithm that estimates the appropriate aperture of the mouth according to the entropy value of the audio stream provided by the TTS system [45].

The Gestures compoNet is in charge of the movement of the robot arms, which can be used for pointing to a specific place or object or to strengthen HRI. Furthermore, combining the entries of Expressions and Conversational, THERAPIST generates expressions adapted to the context, sometimes mimicking and other times creating a different gesture.

The PELEAComp compoNet includes all the modules required to generate and monitor the plan at a symbolic level, as has been previously described.

### Evaluation Outcomes

#### Overview

The THERAPIST project is currently in a preliminary phase of development. We changed the arms of URSUS to increase the robustness and also its face to increase the expressivity. But the main change was the use of RoboCog. The aim is that the robot can play with children in a fully autonomous way and that children experience a high degree of social engagement with the robot. Then, the scenario implies that the practitioner does not need to teleoperate the robot speech or action. There exists, however, a human supervision of the whole session. RoboCog endows the robot with advanced functionalities for interaction. In the next sections, we provide an individual evaluation of the main modules of RoboCog involved in the child-robot interaction (Conversational, Emotions, and Person compoNets), and a preliminary evaluation of how children experience the robot and play with it. This last evaluation uses different types of subjective and objective methods (questionnaires and video analysis) to measure how the children perceive the robot (eg, social presence, emotional attachment) and how the robot interacts with the children.

#### Individual Evaluation

##### Conversational

This compoNet is in charge of processing natural language that supports the child-robot interaction. As mentioned, this processing follows the classical pipeline model including speech recognition (transcription generation and comprehension), natural language generation, and TTS synthesis. The results of speech recognition for five interaction sessions can be seen in Table 1. Each row shows the number of sentences and words for a given session.

**Table 1:** Results of automatic speech recognition.

Experiment	Sentences	Words	Correct words	Percentage of words correctly recognized
#1	8	42	33	78.5
#2	6	34	26	76.4
#3	7	42	32	76.1
#4	12	60	49	81.6
#5	9	59	50	84.7
Total	42	237	190	80.1

It should be noted that the sentences are always related to a topic known by the robot. Therefore, the robot is able to identify the majority of the questions. When it does not understand a sentence, it asks for a second repetition. Once the robot recognizes a sentence, the dialogue is managed as the selection of a response from a database. This database has been built from transcribed dialogues.

##### Emotions

A population of 20 people (10 boys and 10 girls) was recruited for validating the Emotions compoNet. The conductor asked the children to perform sequences of facial expressions where each sequence should cover five expressions: neutral, happiness, sadness, fear, and anger. Each child performed five random sequences of facial expressions. The correct execution of the sequences was validated online by the conductor. The percentage of correct recognition of each facial expression is shown in Table 2. There are not significant differences on the recognition rates for boys or girls.

**Table 2:** Percentage of facial expression recognition.

Facial expression	Correct recognition (boys), %	Correct recognition (girls), %
Neutral	93	92
Happiness	95	96
Sadness	92	91
Fear	91	93
Anger	93	94

##### Person

The Person compoNet is in charge of detecting and tracking the human in front of the robot. These functionalities are successfully solved in most cases. The compoNet also addresses a second major issue: capturing human motion. Although human motion capture is useful, for instance, in designing augmented-reality (AR)-based games, it is mainly used for evaluating the ability of the patient to perform certain movements. Thus, all data from the session are recorded by the robot, allowing the medical professionals to have off-line visualization of the session. Non-invasive human motion capture (HMC) systems have recently experienced a strong evolution due to the commercialization of new cheap depth sensors. Within our framework, we will use our new proposal for HMC [38] inside the Person compoNet. The main novelty of our proposal is that it evaluates the validity of the obtained poses using a model-based pose generator. This tool complements the human tracker provided by frameworks such as OpenNI or the Microsoft SDK. The proposed system enforces kinematics constraints, eliminates odd poses, and filters sensor noise, while learning the real dimensions of the performer’s body. The system was extensively tested and compared against ground-truth data provided by MaxPRO (Innovision Systems Inc. [46]) online data acquisition and a software device that incorporates a motion capture (MoCap) system with six cameras. Table 3 summarizes the results from more than 1500 frames (see [38] for further details) and shows mean errors and standard deviations of the joints. The proposed HMC works close to the tracking software at the shoulder and hand joints. Significant improvement is obtained for the elbow joint.

**Table 3:** Mean errors and standard deviations of right arm joints (in centimeters).

	Hand	Elbow	Shoulder
OpenNI centroids, mean error (SD)	11.3 (5.3 )	6.7 (2.7 )	2.4 (1.0 )
Proposed HMC, mean error (SD)	11.7 (5.0 )	4.9 (1.7 )	2.5 (1.0 )

**Figure 10:**
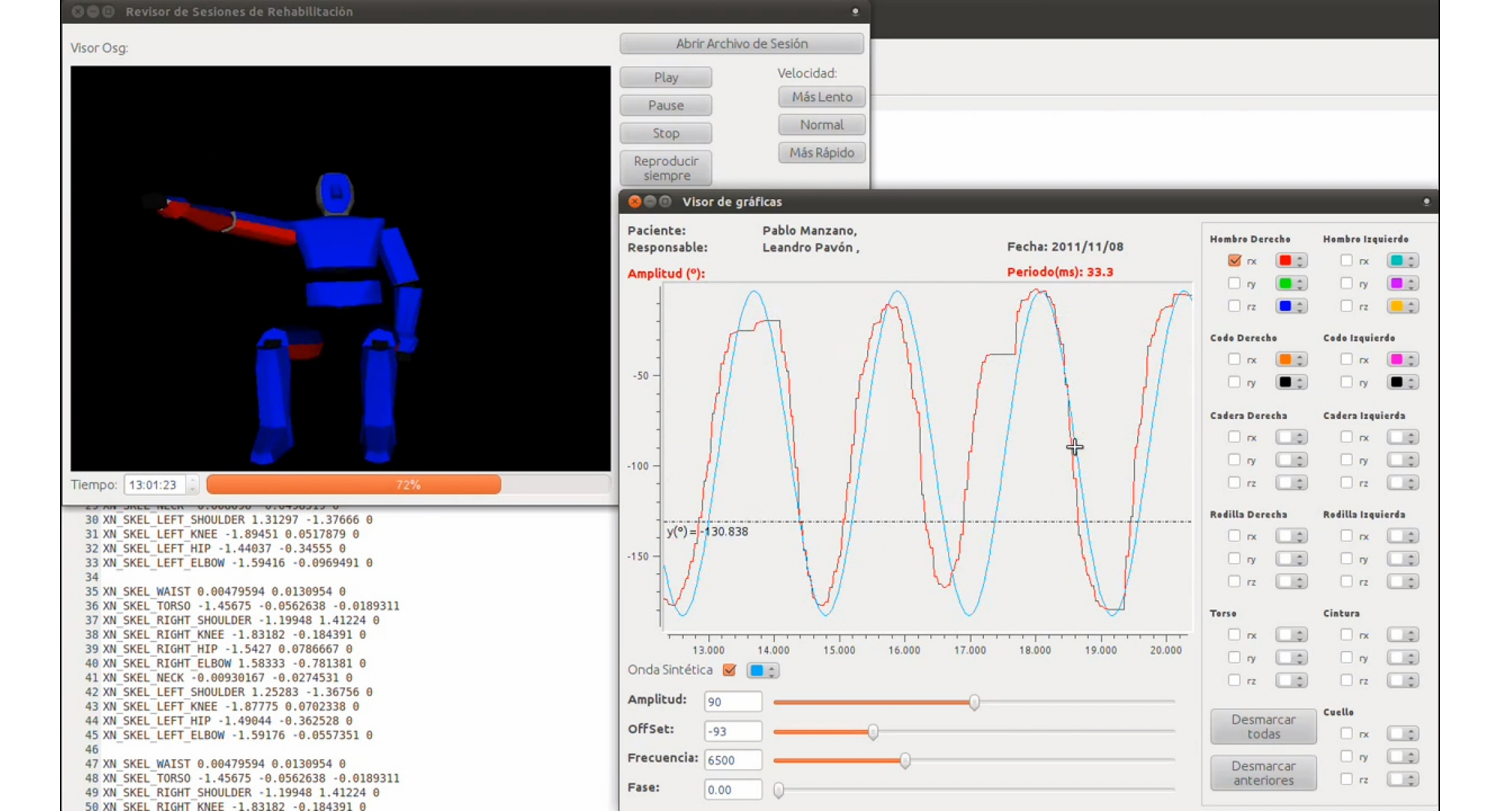
A snapshot of the GUI for off-line visualization and monitoring of the therapeutic session.

The HMC is used for off-line monitoring of the patient’s movements. To do that, recorded data are displayed using a graphical user interface (GUI) that not only provides the video sequence but also numeric information about the amplitude of the movements. Figure 10 shows a snapshot of the GUI.

#### Evaluation With Integrated Modules

##### Overview

Although THERAPIST has not been evaluated in a long-term experiment yet, we have designed simple games where we can measure the ability of the architecture to drive the robot. We describe here one experiment conducted with 35 children. The aim was to evaluate the ability of RoboCog to allow a robot to play with children without supervision. Additionally, we evaluated the social presence of the robot and its ability to encourage children to make certain movements within the play session. Below we provide a detailed description of this experiment.

##### Design and Procedure

**Figure 11:**
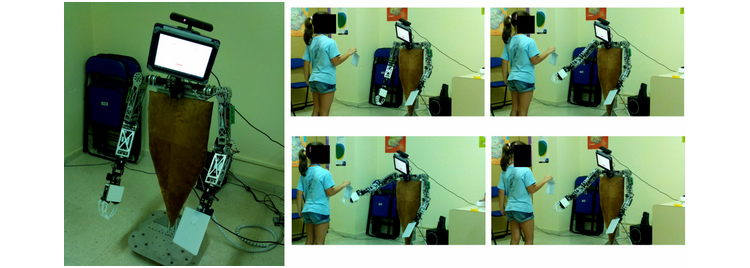
A simple game between a human and a robot: the "touch the draw" game.

This example consists of a simple game between a human and a robot. The robot introduces itself and asks the child to play the game. Upon acknowledgement, the child shows a marker to the robot and it starts to track it, continuously fixing its gaze upon the marker using the RGBD sensor placed in its front-head. After a verbal indication, the robot reaches the marker with its end-effector and waits for a new interaction, or moves its arm back to a resting position after some courtesy delay. The robot tracks the object with its eyes and hand and encourages the child to move the marker up or down, or sideways, during the whole span of the game. It also asks the child to hide the marker. Figure 11 shows some snapshots of one trial. The robot does not wear any specific casing. The mechanical aspect could bias the children’s perception of the robot toward an artificial entity rather than toward a social entity.

From a technical point of view, the development of this interaction game involves several problems such as generalized inverse kinematics, RGBD object detection and tracking, speech recognition and synthesis, and sequential task execution. All these issues have been solved by the RoboCog architecture (as previously described), except the marker detection and tracking, which is solved using a specific compoNet that envelopes the AprilTags visual fiducial system [47]. From the therapeutic point of view, the exercise asks the patient to move the upper limb that holds the marker to avoid the robot’s end-effector. This process is encouraged by the robot through verbal sentences. The movements are always performed in front of the robot, so the motion capture can be achieved with precision. The distance to the robot is also reduced.

##### Participants

The game was initially validated in the research lab and then tested with children within the target population (children ranging 4-9 years old). Briefly, the aim was to test the child-robot interaction and to propose the character of our robot as believable. This experiment was carried out in July 2014. The overall population consisted of 35 children (16 girls and 19 boys). The mean age was 7.37 years old (SD 1.47). The participants were recruited from a summer school. The leader, who was a teacher familiar to the child, introduced the game to the child and brought him/her to the play area with only the instruction to play with the robot for a while. In the play area, the robot introduced itself and then the game started. Sessions were recorded, and video coding was done after the sessions. However, the robot-child interaction was mainly analyzed through a questionnaire, which was given immediately after having played with the robot. A second questionnaire evaluating the robot’s behavior was filled out by an external observer.

##### Hypothesis

Our starting hypothesis is that pediatric patients would get consistently engaged in a therapeutic non-physical interaction with a robot. In the short term, this will facilitate the design of new therapies that should improve the patient’s motivation to address the tedious rehabilitation therapies. However, in this work, we focus on the verification of this preliminary hypothesis. This study analyzes the more specific predictions that we expect the children to see the robot as a social entity and not an artificial one, and that we expect the children to enjoy playing with the robot and would like to repeat the experience.

Both issues are basic to establishing a social bond between the robot and the child, which allows the robot to help the child successfully achieve long-term rehabilitation therapies. Additionally, to drive this effective interaction, the robot must be able to emanate responses at human rates. Therefore, we also aim to verify a second hypothesis, that exploiting the RoboCog possibilities, THERAPIST engages the child in an active interactive process.

##### Measures and Metrics

Experimental sessions were video recorded and, when the playtime was over, the leader told the child that the session was over and instructed them to answer some questions about the session (Questionnaire Q1). An external observer filled out a second questionnaire, Q2. Questionnaire Q1 was adapted from previous work from Heerink et al [48], Weiss et al [49], and Bailenson et al [50]. Questionnaire Q2 was adapted from the work of Joosse et al [51]. The aim of both video analysis and questionnaires was to verify our stated hypotheses. We know from previous work that children enjoy playing with robots and that they endow these robots with cognition or feelings. We must, however, verify if this is the case for THERAPIST, which will work in an unsupervised way.

Questionnaire Q1 tries to verify if the children perceive the robot as a social entity rather than as an artificial or teleoperated machine. Several dependent variables were measured (social presence, emotional attachment, etc) related to the verification of our first hypothesis. Different types of questions were posed in the questionnaire. Social Presence was assessed using five questions, derived from a questionnaire adapted from Heerink et al [48]. These questions featured statements responded to on a 5-point Likert scale. Social versus Artificial perception was evaluated using the attribution of adjectives to the robot [52]. A list of 20 adjectives was provided to the child who must choose an initial set of 3 for describing the robot (List 1), and then another 3 adjectives different from the first set (List 2). On the list of 20 adjectives, we added 10 referring to a social entity (clever, angry, stupid, patient, etc) and 10 referring to an artificial one (useful, simple, solid, etc). Individual scores were assigned to each chosen adjective: +2 points and +1 point to “social” adjectives on the Lists 1 and 2 respectively, and -2 and -1 to “‘artificial” adjectives on Lists 1 and 2. The final score for the variable was calculated as the arithmetic sum of scores assigned to the chosen adjectives.

The cognitive abilities that children assigned to the robot (Cognition) were assessed through two questions. Each question elicited a yes/no answer, reflecting the child’s affirmation or negation. Questions were adapted from [49]. Emotional attachment was evaluated through two direct questions (answered with a yes/no response) and through two additional questions responded to on a 5-point Likert scale. Social reciprocity was assessed through three questions, responded with a yes/no answer.

The evaluation of these questionnaires implied first the measure of their internal consistency using Cronbach alpha. This statistic measures how well a set of variables or items measures a single, one-dimensional latent aspect of individuals. It is appropriate for our case as we cannot measure explicitly the variables; we asked a series of questions and combined the answers into a single numerical value. Then, the evaluation of the expected predictions related to our first hypothesis was performed by estimating the mean and standard deviations of the obtained scores. Furthermore, dependent variables (Pearson correlation analysis) were correlated to analyze the dependence among them. Age and gender factors were considered.

The quantitative evaluation of the video data allows evaluation of the emotional response/interactive behavior of the children (our first hypothesis) and the annotation of the video sequences through systematic observation. A coding scheme was built to evaluate the children’s response (occurrence and amount of time spent on the predefined key behaviors/emotional state). The coding scheme is summarized in Table 4. Category encoding allows description of the subject in terms of meaningful activity. The categories Reciprocity, Affection, and Artifact were taken from Melson et al [53]. We added Playing (the child is immersed in the game). When the child was in front of the robot but looked/spoke to the leader, we considered that the child took the robot as an artifact. Several behaviors could be categorized as Affection, Reciprocity, or Playing. In these cases, the first two categories were prioritized. The whole coding scheme was adapted from [48]. Several behaviors are difficult to categorize. Videos were annotated by two observers to increase the reliability of the manual coding. Results (time duration) were provided as the mean values from both observers.

Finally, the robot’s behavior and attitude (our second hypothesis) were evaluated using the questionnaire Q2, which responds to a model similar to that used by Joosse et al [51] to generate the database BEHAVE-II. The questionnaire was designed to be filled in from the point of view of the person observing the behavior of the user against the presence of the robot. In this sense, it is influenced by the Almere original model and other previous work on man-machine interaction. In particular, the questionnaire includes a collection of questions arranged in four blocks (robot motion, conversation, interaction, and general sensations). Cronbach alpha was again used to measure the internal consistency of the tests. The evaluation of the expected predictions related to our second hypothesis was again performed by estimating the mean and standard deviations of the obtained scores.

**Table 4:** Coding scheme for video annotation.

Group	Behavior	Analytical category
Emotions	Enjoyment, Boredom, Frustration, Neutral, Fear	
Verbal	Speak to the robot	Reciprocity
	Speak to others about the robot	Artifact
	Speak to others	Artifact
Distance	Maintain the social distance	Reciprocity
	Moves away from the robot	Affection (negative)
	Moves toward the robot	Affection (positive)
Gaze	Eye-contact	Reciprocity
	Look at the robot / marker	Playing
	Look at others	Artifact
Play	Help the robot to touch the marker	Affection (positive)
	Hides the mark	Playing

##### Questionnaire Q1 Results

To evaluate the reliability of the questionnaires, Cronbach alpha was used to measure their internal consistency. As Table 5 shows, obtained values can be stated as correct. Furthermore, values are not excessively high, which could suggest that we are asking the same question in slightly different ways.

The alpha value for the Emotional attachment is <.7 (considered as an indication of a reliable construct). As mentioned, this variable was evaluated through two yes/no questions (“Do you think that the robot can see/hear you?”) and two 5-point Likert scale questions (“Would you like to have a robot at home?” and “When you are at the hospital, would you like to be attended by a robot?”). This last question was evaluated by the children with low values. Clearly, they like to be attended by a familiar or friend when they are at the hospital.

**Table 5:** Cronbach alpha values.

	Cronbach alpha
Social presence	.758
Cognition	.714
Emotional attachment	.616
Social reciprocity	.808

Table 6 illustrates the results of Pearson correlation analysis. It shows that the score on Social presence correlates with the Emotional attachment, Cognition, and Social reciprocity variables. Contrary to the work of Heerink et al [48], all variables (except Social vs Artificial perception) show a positive correlation with Age, that is, the experience of a social entity is strongest for older children. It should be noted, however, that our participants were aged between 6 and 9 years, whereas participants were aged between 6 and 12 years in Heerink et al’s work. Furthermore, the robot used in the Heerink et al’s work was a PLEO one, very different from our platform (see Figure 11). It will require a deeper study to analyze when the children starts to perceive the robot as a social or an artificial entity, and the degree of importance that the robot’s appearance has on the evaluation of these variables. On the other hand, Emotional attachment shows a positive correlation with gender, indicating that this variable is strongest for girls rather than for boys. There was not a clear correlation between gender and any other variable. It should be noted that, within our participants, there was not a correlation between Age and Gender (Pearson’s *r*=.050).

**Table 6:** Correlation (Pearson’s *r*) of questionnaire Q1 items.

	Social presence	Cognition	Emotional attachment	Social reciprocity	Social vs artificial perception
Age	.350, *P*<.05 (two-tailed)	.303, *P*<.10 (two-tailed)	.437, *P*<.05 (two-tailed)	.301, *P*<.10 (two-tailed)	-.019
Gender	.133	.110	.350, *P*<.05 (two-tailed)	.136	.135
Social presence	1.000	.583, *P*<.01 (two-tailed)	.399, *P*<.02 (two-tailed)	.360, *P*<.05 (two-tailed)	-.072

The Social versus Artificial perception was not correlated with any other variable. However, children tended to categorize the robot using “social” adjectives. Thus, on List 1 from the 35 participants (105 adjectives), the most used adjectives were “Loving” (19), “Friendly” (16), and “Polite” (13). The first “artificial” adjective on this set was “Well designed” (13). Other used adjectives were “Clever” (12) and “Nice” (10), which are both “social” adjectives. Table 7 summarizes the scores (mean and standard deviations) assigned to the different questions on Q1. There was good acceptance of the robot among the children. The Social versus Artificial perception was clearly biased toward “social” entity.

**Table 7:** Mean and standard deviations of questionnaire Q1 items.

Q1 items	Mean	SD
Social presence: 5—positive, 0—negative	4.102	0.764
Cognition: 1—positive, 0—negative	0.900	0.265
Emotional attachment: 2—positive, 0—negative	1.717	0.396
Social reciprocity: 1—positive, 0—negative	0.952	0.183
Social vs Artificial perception: +9—social, -9—artificial	3.514	3.337

##### Video Data

Participants are not usually familiar with robotics. Videos show that children are initially shy and find it strange to be playing with a robot. They do not speak, and although they are looking at THERAPIST and follow its commands, they do not show a reciprocity response to the robot’s presence. This situation becomes more relaxed when the robot speaks to them, asking for the child’s favorite football team or when it tells them that they are playing very well. Typically, the child replies to the robot’s questions and then begins to become more active in the game. In 1-2 minutes, the child typically starts to have fun, moves the marker in front of the robot so that the robot does not reach it, hides the marker, or if they see that the robot fails to reach it for some time, they move the marker to the robot’s end-effector to help the robot to win. Thus, children exhibit a final positive response to the robot: they smile or the leader must ask them to finish up several times as they continue playing.

**Figure 12:**
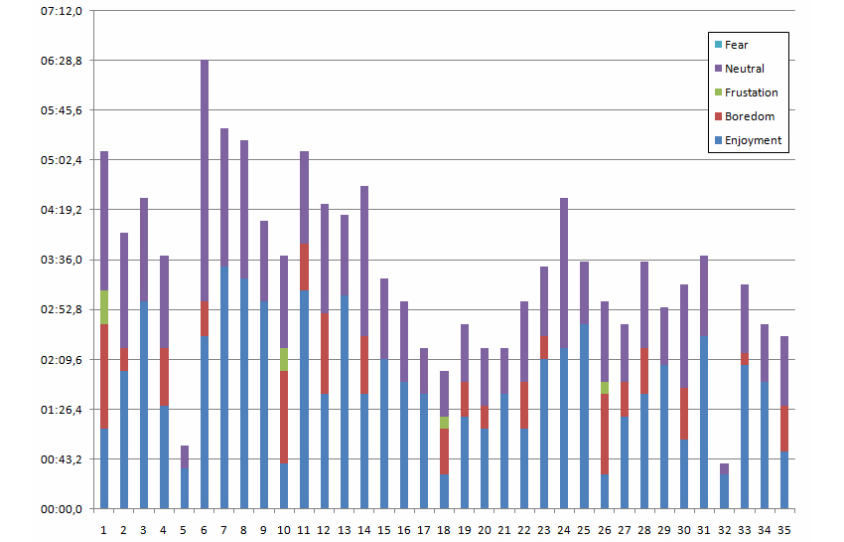
Emotional states of the children during playing time (in seconds).

Each session usually took 3-4 minutes. After analyzing the video sequences, the emotional state of each child was manually annotated for the whole session. Figure 12 summarizes the results. It can be noted that neutral and enjoyment were the most common states, but also that 3 children found the experience especially boring (subjects #1, #10, and #26). The boredom is mainly present at the beginning of the session, and as mentioned, disappears when the robot touches the marker several times and the robot and child then start to have a conversation.

**Figure 13:**
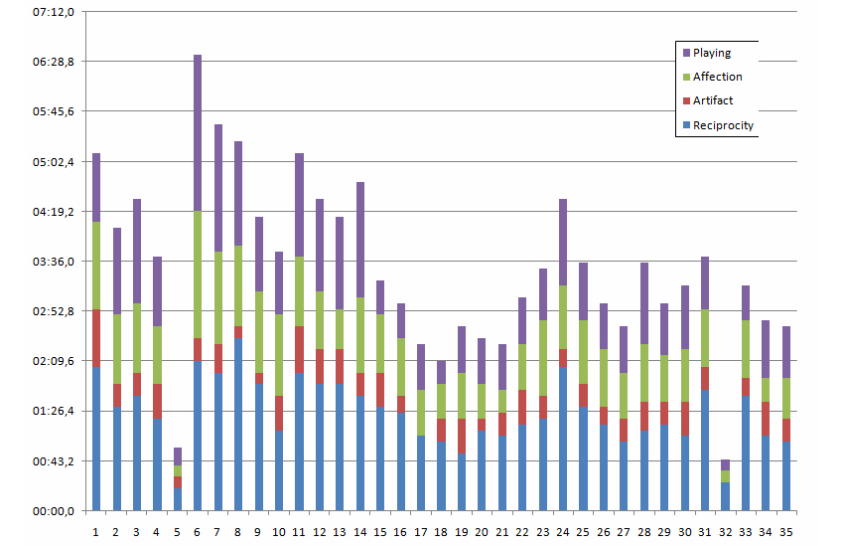
Playing time distribution (in seconds) among modality categories: Affection, Reciprocity, Artifact, and Playing.

The time distribution (in seconds) among the modality categories of the playing time for each children is illustrated in Figure 13. It should be noted that the total time for each session does not exactly match with the time in Figure 12 or 13, as the initial presentation, where the leader was with the child was not considered. There are also some time intervals that cannot be clearly categorized (the two observers do not agree), and they were also removed. As mentioned, for Figure 13, there are two main categories: Artifact, where the child looks at the leader or at the robot (but not at its face) and speaks to the conductor, and Playing. Affection and Reciprocity appear during playing (ie, they can be considered as subcategories within Playing). Figure 13 shows that all children were mainly playing with the robot. Typically, the Artifact label appeared at the beginning of the sessions.

##### Questionnaire Q2 Results

Questionnaire Q2 consists of four blocks, each one of which includes two questions. These questionnaires evaluated the robot’s behavior and the social correctness (speed of response, natural motion, etc) of the interaction. Questions were answered on a 5-point Likert scale. Cronbach alpha values show that questions within each block were consistently answered (all values are over .7). Table 8 summarizes the results, from which we conclude that the interaction between the robot and the child was usually fluent, and all channels (verbal and non-verbal) were appropriate. It should be noted that the robot does not get blocked during the sessions.

**Table 8:** Mean and standard deviations of questionnaire Q2 items.

	Mean	Standard deviation
Are the robot’s movements natural?	4.66	0.40
Has the child stepped away from the robot?	0.23	0.55
Have you understood what the robot told the child?	4.76	0.43
Does the robot understand the child?	4.20	0.72
Does the robot get blocked?	0.00	0.00
Was the interaction fluent?	4.00	0.69
Do you think the child enjoyed the experience?	4.52	0.56
Do you think the child wants to repeat the experience?	4.41	0.55

##  Discussion

### Principal Results

Rehabilitation robotics constitutes an emerging area of research, where the aim is to include robotics technology in the time-consuming and labor-intensive process associated with neurorehabilitation therapies. As in other fields of application, robots can offer several key advantages, such as the possibility of performing a consistent and personalized treatment without tiring, and its capacity to use sensors to acquire data, which can provide an objective quantification of the recovery. Moreover, apart from giving mechanical/physical assistance in rehabilitation, recent studies postulate that robotic technology can motivate and coach patients in the realization of the repetitive efforts that constitute the primary stimulus for recovery. Due to these reasons, one of the more active research challenges in rehabilitation robotics is the design and development of safe and effective human-robot interaction for hands-off, socially assistive robotics. Clinical experiments demonstrate that motivation is an important factor for successfully addressing a lengthy neurorehabilitation therapy, and it is usually employed as a determinant of rehabilitation outcome. Hence, active engagement towards a therapy is typically equated with motivation. Within this context, socially assistive robots emerge as a new field of robotics whose aim is to develop systems that assist patients through social rather than physical interaction. They provide therapy oversight, coaching, and motivation using the robot’s abilities to interact and maintain the interest of patients. Furthermore, depending on the degree of autonomy of the robot, coaching and motivation can be provided with little supervision by professional therapists. It should be noted that there is typically little use in developing a teleoperated assistive robot for working at the hospital, except for some specific use cases. Telerobotics is usually restricted to providing an effective remote supervision of the therapy at home after hospitalization or for surrogate clinicians passing medical consultations. This paper describes a novel cognitive architecture, which fulfils the requirements needed by a socially assistive robot used as therapist assistants in real hospital rehabilitation scenarios.

Currently, the validation methodology considers only metrics related to the social presence of the robot and the human-robot interaction. These metrics quantified that the level of attention and engagement between robot and child were positive. Quantitative and qualitative results (obtained from several polls of the participants in the experiments: children, parents, and technical and medical staff) showed that the patients enjoyed the sessions, and they considered them more fun and motivating than using only the conventional treatment. Moreover, the medical staff also considered the rehabilitation session positive for the children’s rehabilitation process, and the results recorded by the robot very useful for analyzing the patients’ progress and for planning personalized future rehabilitation sessions. Briefly, it can be concluded that the robot was able to achieve a high level of engagement with the patient, maintaining their levels of motivation and adherence to the therapeutic session.

### Limitations

In order to correctly evaluate the validity and benefits of this proposal, future work will focus on testing our proposal in a long-term clinical use case conducted at the Hospital Universitario Virgen del Rocío (Seville) with pediatric patients with upper limb motor deficit due to obstetric brachial plexus palsy and cerebral palsy. Both cases are among the most prevalent pathologies causing motor and cognitive deficits. Pediatric patients represent an interesting collective that are favorable to these systems, as recent pioneering studies, including our own work, have shown that they can be easily driven into highly attentive and collaborating attitudes by letting them interact with social robots. The clinical variables that will be used for evaluating the clinical evolution of the patient will be passive and active articular balance of the shoulder, elbow, and hand; the degree of concordance (ie, precision of the movements performed by the child with respect to theoretical values); motor function of upper limbs; and patient satisfaction.

Restricted to well-defined environments, the evolution of our proposal should be followed from the study of therapeutic sessions of increasing difficulty attending to the autonomy of the robot. From these pilot experiments, we will iteratively evaluate and improve the functionalities of THERAPIST, following a scheme requiring the active participation of engineers, therapists, and patients within the loop, not only as designers or evaluators, but also as interactive partners. This evaluation will also require the definition of metrics and monitoring protocols to evaluate the progress of the patients, and comparisons of the results with classical therapies.

### Comparison With Prior Work

With respect to the state-of-the-art, the cognitive architecture of THERAPIST provides original contributions that, consequently, influence the therapeutic process. The first is the construction of a deep representation that stores and updates both situational and symbolic data at several levels of abstraction. The lowest level of representation constitutes a non-homogeneous, crude/raw representation of the robot’s environment (including people and the robot itself). The highest level of the hierarchy provides a graph grammars-based description easily usable by planning and searching algorithms.

Second, the learning and robot behavior adapts in an open-ended way as a function of the user profile (eg, emotional and physical response to the required movements) in order to provide a customized interaction with the users while achieving a cooperative task. To this end, the whole architecture grounds high-level and mid-level responses through the Executive module.

Third, our proposed multimodal interaction process aims to determine the user’s state as well as to produce the adequate response. This process involves verbal and non-verbal channels and includes the human response in the loop. Hence, beyond traditional interaction technologies such as user localization, tracking, speech and gesture recognition [54], RoboCog incorporates methods that allow THERAPIST to show an affective behavior through the recognition of facial emotional states and the generation of expressive expressions and/or gestures. These are all fundamental elements for initiating, maintaining, and regulating interaction with the child. In the therapy, all referred procedures are used to ensure a continuous interaction.

Fourth, non-verbal dialogue between the robot and the human supports effective and dependable robot behaviors. This leans heavily on the evaluation of those robot non-verbal behaviors that impact trust and acceptance: detection of the human state and real-time execution and adaptation of the robot’s behaviors.

Finally, planning and plan execution will be treated in an integrated, heuristic approach that allows for hierarchical abstraction together with reactive decision making.

### Conclusions

Within a general framework of hands-off robotics rehabilitation, this paper describes the general principles of design and the concrete instantiation of the software architecture of THERAPIST. The RoboCog architecture allows THERAPIST to synchronize the generation and/or recognition of verbal and non-verbal interactive cues. It also allows the robot to merge at the same time, high- and mid-level skills. These features have been evaluated through exercises of increasing complexity. In this paper, we present how the robot is able to play a game with a human user. From a therapeutic point of view, this simple game causes the human to move her upper limbs while speaking with the robot. The use of several channels (voice, facial expressions, and gestures) improves the acceptance of the game by the general public, since the robot is seen as a more friendly and empathetic machine. We consider these features critical to engaging patients in therapeutic sessions.

It is, however, necessary to design and evaluate more complex games if we want to sustain human attention for longer periods of time (eg, a typical therapeutic session takes 20 minutes). This can be done by using additional resources. To this end, we have tested the use of an augmented reality (AR)-based application [55]. Within this application, the perception system of the robot captures the human body as it is used in real-time to include the patient inside the virtual environment. As mentioned, this human motion capture functionality is also the basis for a monitoring tool, which allows clinic professionals to evaluate and monitor the efficiency of the therapeutic sessions.

## Abbreviations

AGM: active grammar-based modeling
AR: augmented reality
ASR: automatic speech recognition
AU: action unit
BoW: Bag of Words
GUI: graphical user interface
HAL: hardware abstraction layer
HMC: human motion capture
HRI: human-robot interaction
ORM: Obstacle Restriction Method
PDDL: Planning Domain Definition Language
RGBD: Color (Red-Green-Blue) and Depth Camera
SDK: Software Development Kit
TTS: Text-To-Speech
UEx: University of Extremadura
